# Supplementary figures and images for: An orally available PfPKG inhibitor blocks Plasmodium’s infection of the liver
Source: PLoS Pathog. 2026 Jul 30;22(7):e1014322. doi: 10.1371/journal.ppat.1014322 (PMC13422867; doi:10.1371/journal.ppat.1014322)

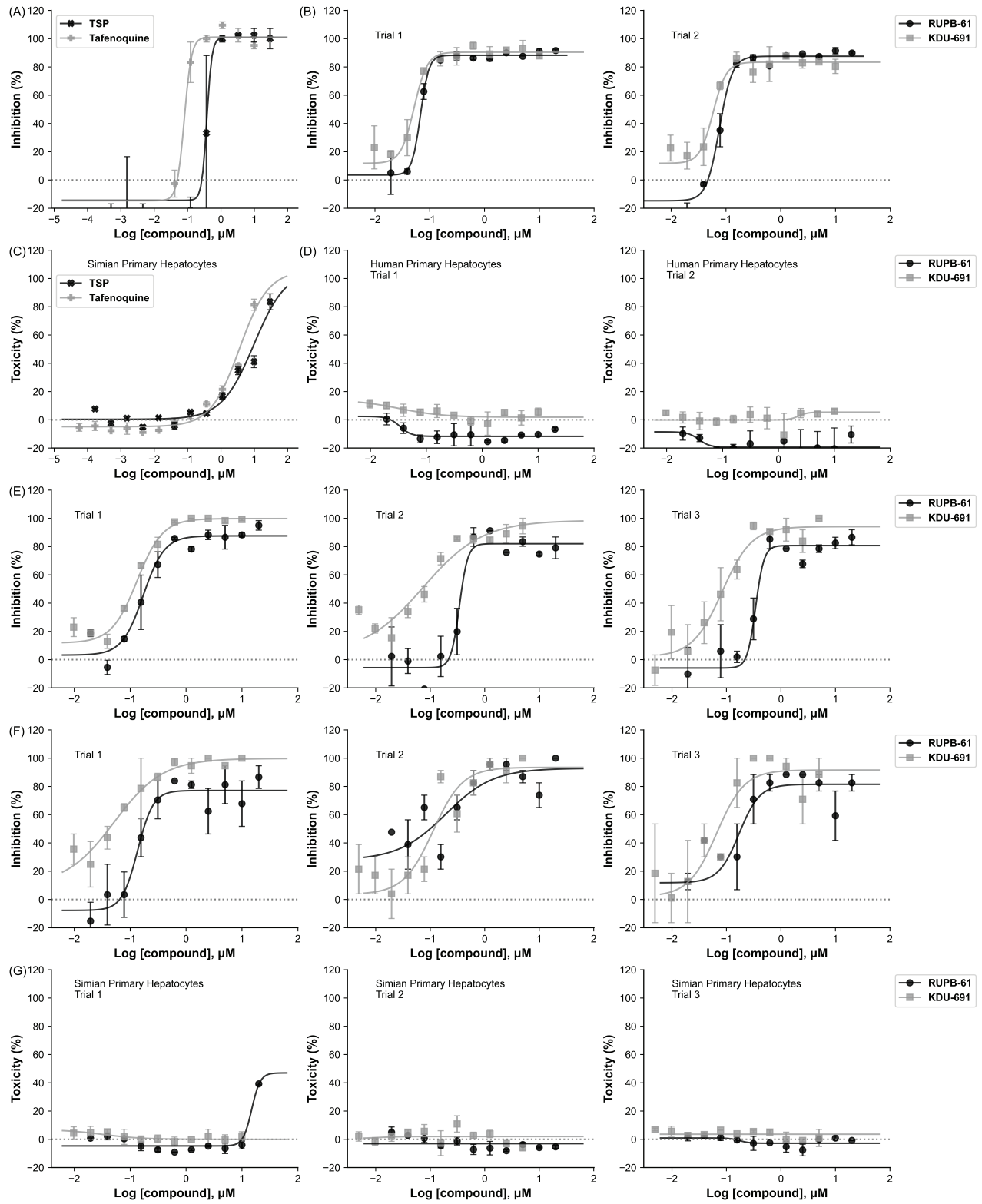

Supplement: S1 Fig — (A) Dose-response curve of TSP against P. cynomolgi hepatic hypnozoites formed in simian hepatocytes. Tafenoquine was used as a positive control. Data shown are normalized to number of infected cells in vehicle control. Data shown are from a single trial performed in technical duplicates. (B) Dose-response curves from two independent trials of RUPB-61 against P. falciparum sporozoites in human hepatocytes. Data shown are normalized to the number of infected cells in vehicle control. KDU-691 was used as a positive control. (C) Effect of TSP on the survival of primary simian hepatocytes. Data shown are from a single biological replicate performed in technical duplicates. (D) Effect of RUPB-61 on the survival of primary human hepatocytes. Data shown are mean of two independent trials performed in technical duplicates. (E) Dose-response curves from three independent trials of RUPB-61 against P. cynomolgi sporozoites in primary simian hepatocytes. Data shown are normalized to the number of infected cells in vehicle control. KDU-691 was used as a positive control. (F) Effect of RUPB-61 on the survival of primary simian hepatocytes. Data shown are mean of three independent trials performed in technical duplicates or triplicates. (PDF) [file ppat.1014322.s001.pdf]

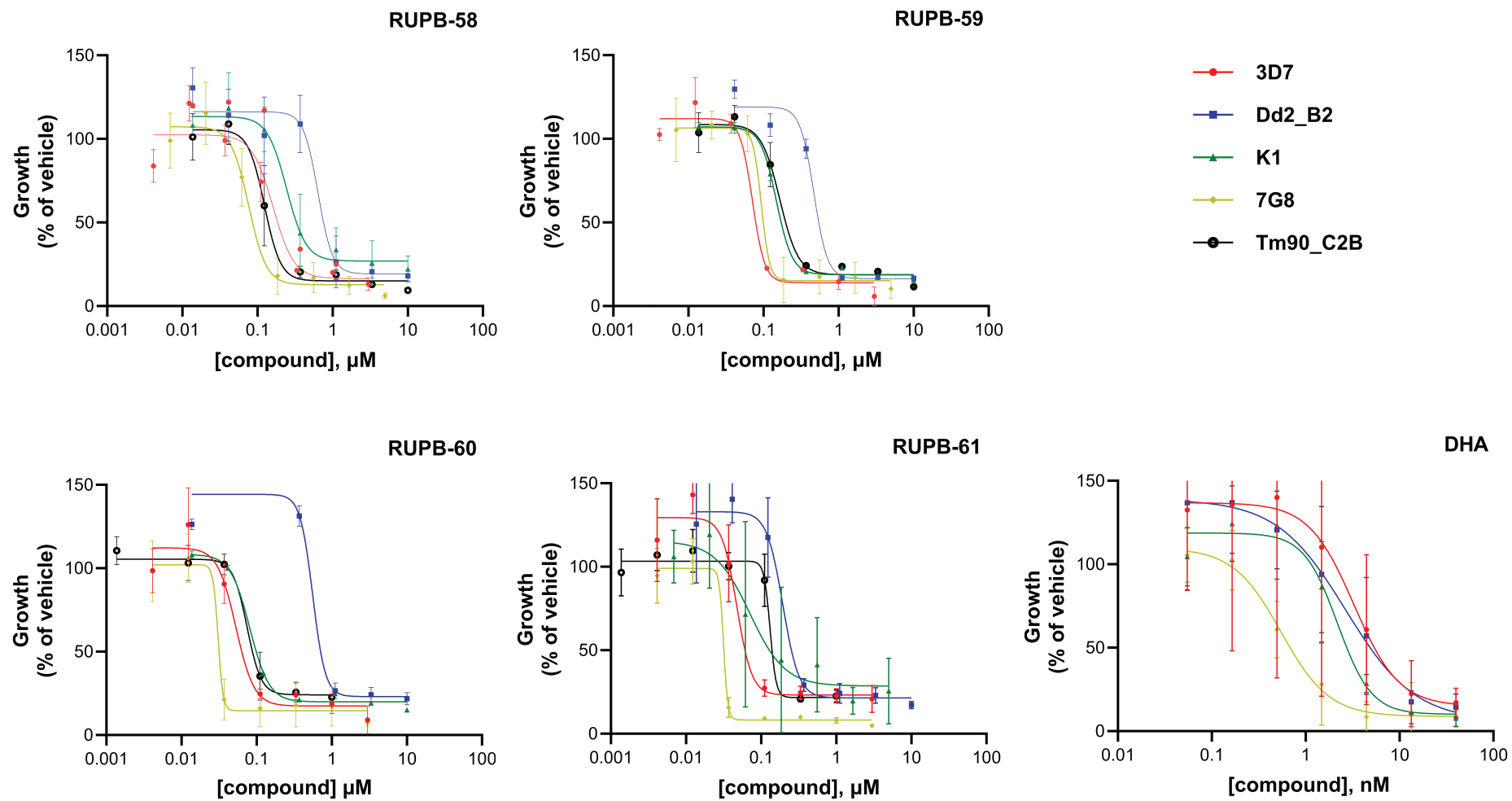

Supplement: S2 Fig — Growth inhibition of 3D7, Dd2-B2, TM90_C2B, K1, 7G8 over 72h in asexual growth assays was measured using SybrGreen incorporation. (PDF) [file ppat.1014322.s002.pdf]

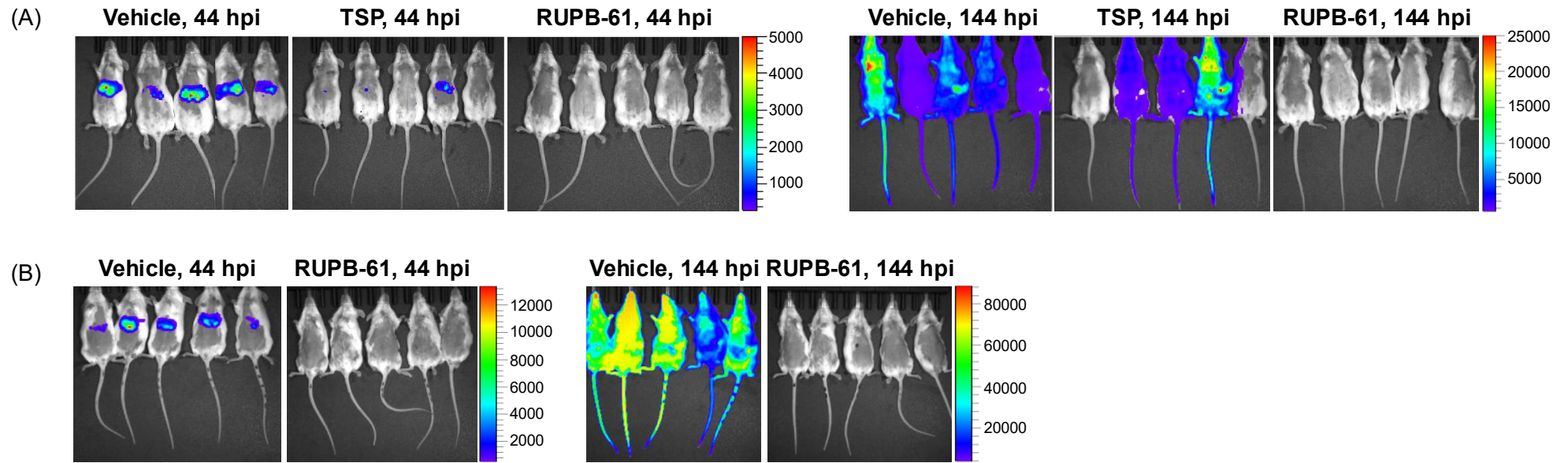

Supplement: S3 Fig — Bioluminescent imaging of mice to quantify liver parasitemia in mice infected with PbLuc-GFP sporozoites. (A) Mice administered three intravenous doses of vehicle or compound. Images were obtained at 44 hpi and 144 hpi. (B) Mice administered three oral doses of vehicle or compound. Images were obtained at 44 hpi and 144 hpi. (PDF) [file ppat.1014322.s003.pdf]

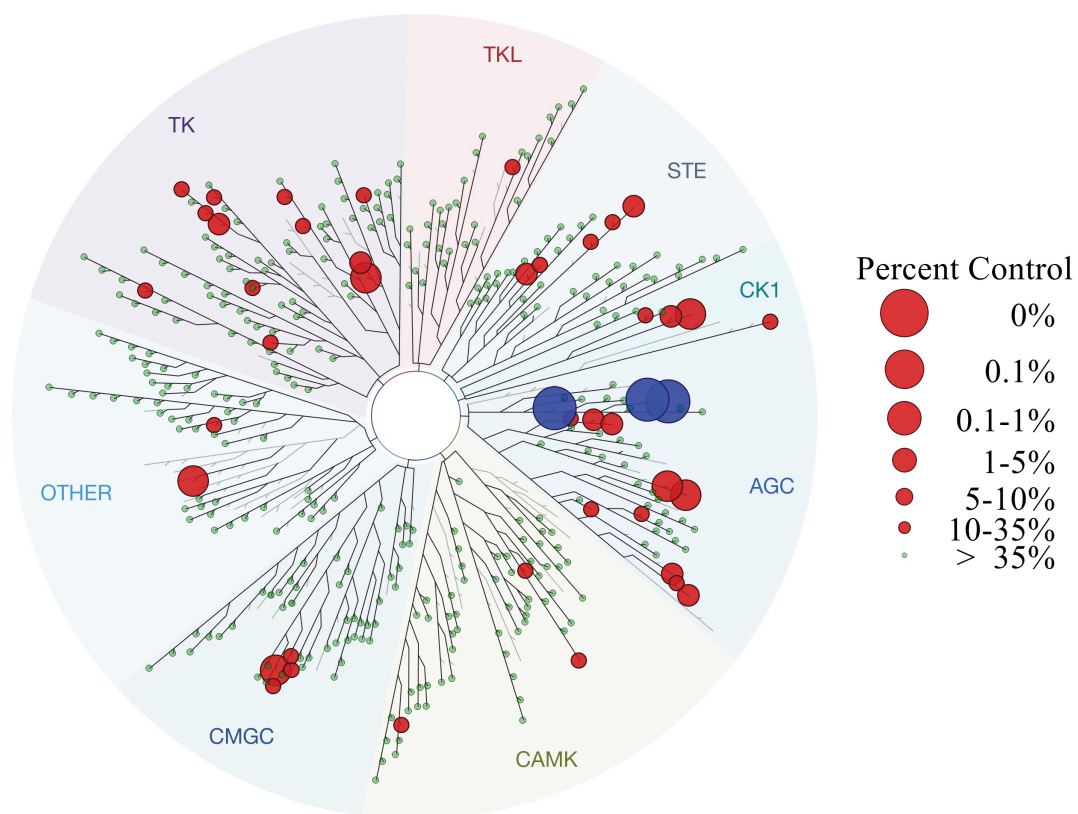

Supplement: S4 Fig — Interaction of RUPB-61 at 1 µM with 400 unique human kinases was determined using a panel of in vitro competition binding assays. The effect of RUPB-61 on the binding of a target kinase to its test probe is reported as a percentage of enzyme binding in the presence of vehicle alone. Blue circles represent the major human off-targets, CIT, ROCK1 and ROCK2. TKL: Tyrosine Kinase-like; STE: Ste kinases; CK1: Casein Kinase 1; AGC: Protein Kinase A, G and C; CAMK: calmodulin kinase; CMGC: Cyclin-dependent kinases, Mitogen-activated protein kinases, Glycogen synthase kinases and CDC-like kinases. (PDF) [file ppat.1014322.s004.pdf]

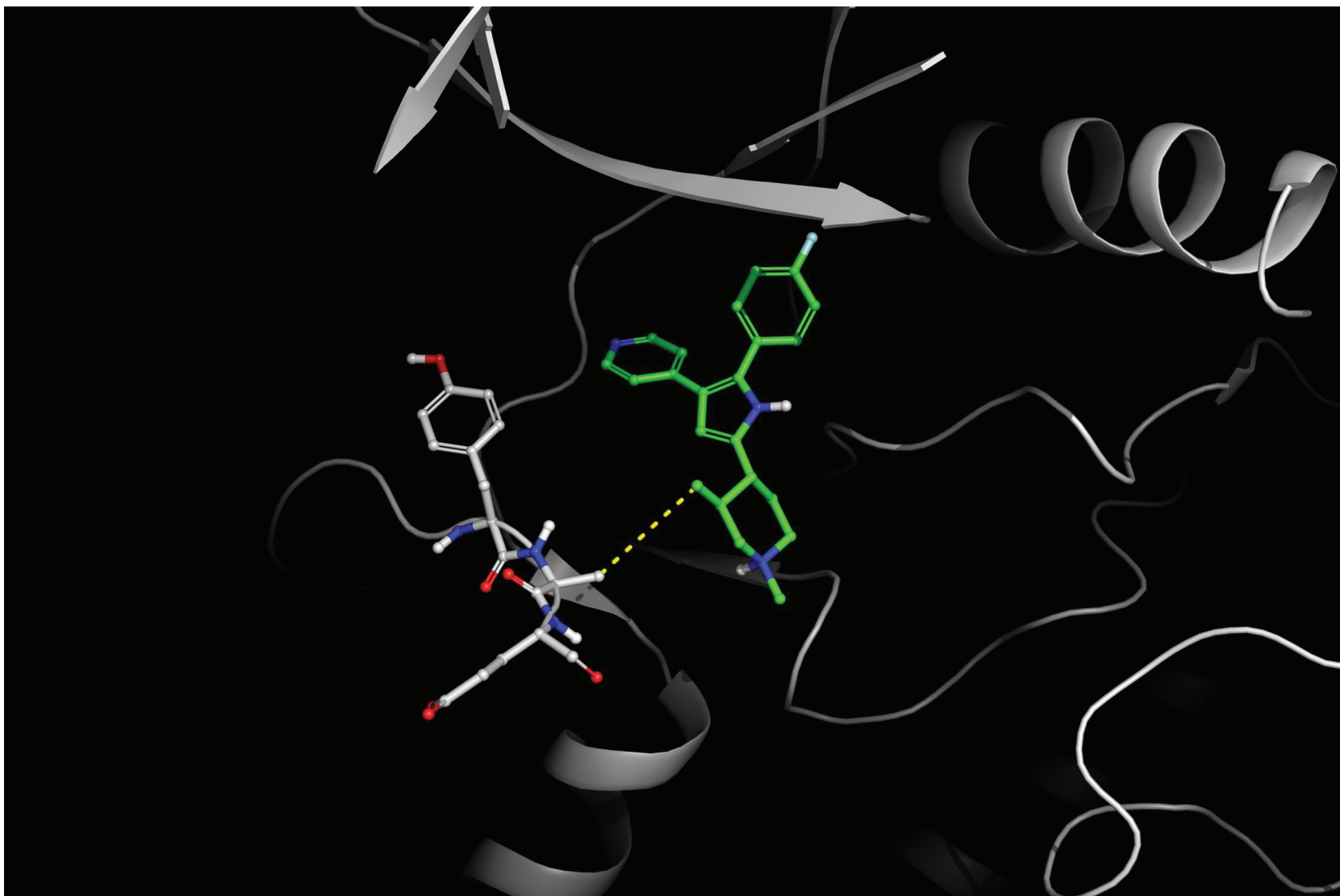

Supplement: S5 Fig — Compared to the structure with RUPB-61, the methyl substituent on the RUPB-60 (green) piperidine evidently leads to the resolution of Ala816 (grey) in the C-term tail (dotted line) (Ala823 in PfPKG). (PDF) [file ppat.1014322.s005.pdf]

**A**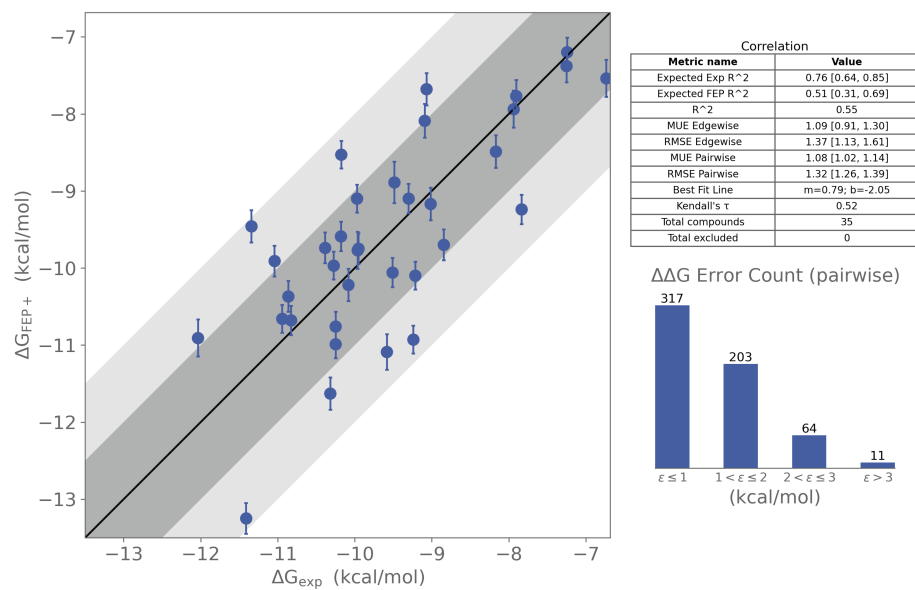**B**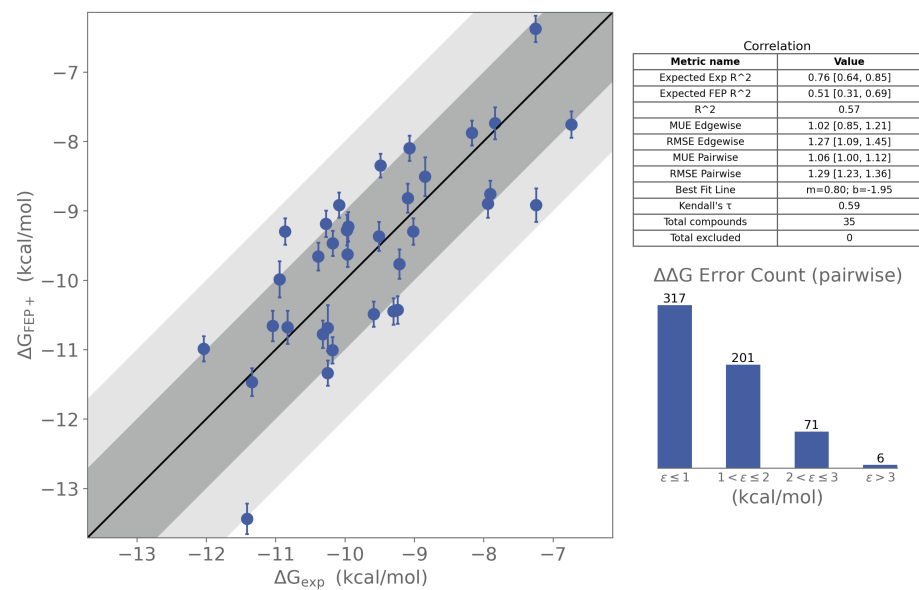

Supplement: S6 Fig — A congeneric set of 35 ligands was used in the (A) IFD-MD model of PfPKG (B) experimental PvPKG structure. (PDF) [file ppat.1014322.s006.pdf]
